# High-Resolution Mapping of Anthropogenic Heat in China from 1992 to 2010

**DOI:** 10.3390/ijerph110404066

**Published:** 2014-04-14

**Authors:** Wangming Yang, Bing Chen, Xuefeng Cui

**Affiliations:** 1 State Key Laboratory of Earth Surface Processes and Resource Ecology, College of Global Change and Earth System Science, Beijing Normal University, Beijing 100875, China; E-Mail: yangwm2012@gmail.com; 2 State Key Laboratory of Remote Sensing Science, Institute of Remote Sensing and Digital Earth, Chinese Academy of Sciences, Beijing 100101, China; E-Mail: chenbing@mail.iap.ac.cn

**Keywords:** anthropogenic heat, urban heat island, DMSP-OLS, major metropolis

## Abstract

Anthropogenic heat generated by human activity contributes to urban and regional climate warming. Due to the resolution and accuracy of existing anthropogenic heat data, it is difficult to analyze and simulate the corresponding effects. This study exploited a new method to estimate high spatial and temporal resolutions of anthropogenic heat based on long-term data of energy consumption and the US Air Force Defense Meteorological Satellite Program-Operational Linescan System (DMSP-OLS) data from 1992 to 2010 across China. Our results showed that, throughout the entire study period, there are apparent increasing trends in anthropogenic heat in three major metropoli, *i.e.*, the Beijing-Tianjin region, the Yangzi River delta and the Pearl River delta. The annual mean anthropogenic heat fluxes for Beijing, Shanghai and Guangzhou in 2010 were 17 Wm^−2^, 19 and 7.8 Wm^−2^, respectively. Comparisons with previous studies indicate that DMSP-OLS data could provide a better spatial proxy for estimating anthropogenic heat than population density and our analysis shows better performance at large scales for estimation of anthropogenic heat.

## 1. Introduction

Anthropogenic heat is wasted heat that is released into the atmosphere in sensible and latent forms [1]. It is released mainly through space heating and cooling, running appliances, transportation, industrial processes, and human metabolism [2,3,4,5]. Nearly 70% of energy is consumed within cities that occupy only 2% of the Earth’s surface area [6]. Therefore, the magnitude of the anthropogenic heat flux (AHF) in urban areas is much larger than that in rural areas. AHF has high spatial and temporal variations which are sensitive to climate zone, population density, economic development, season, time of day, and urban form or function [7,8,9]. In general, it is larger in winter than in summer, in downtown than in suburbs, in cold climates than in warm climates, in developed areas than in developing areas, in big cities than in small cities. AHF has been suggested as an important contributor for local urban warming [10], annual mean warming across western European [11], and even global surface warming through disruption of atmospheric circulation patterns [12].

AHF can be calculated using either observation or inventory approaches. Observations include surface energy budget measurements [13,14,15] and *in situ* eddy covariance observations [16]. Currently, inventory approaches (bottom-up or top-down) are more widely used to estimate AHF from global scale to single city [8,17]. The top-down approach is mapping energy consumption from coarse spatial and temporal resolutions to higher spatial and temporal grids [3,9,18]. While the bottom-up calculates detailed statistics of the sub-system, *i.e.*, time and volume of traffic, building height and population density of the entire studied urban area [19]. The first global AHF data was released in 2009 (referred as Flanner study hereafter), and used the top-down inventory method to apportion non-renewable energy consumption in a 2.5-min spatial resolution (about 4–5 km at the equator) mainly based on global map of population density [20]. Flanner study shows unreliable information for some regions such as China, as spatial population density is not a good proxy for economic development or energy consumption in China [21]. The nighttime light data from the US Air Force Defense Meteorological Satellite Program-Operational Linescan System (DMSP-OLS) has been found to be highly correlated with AHF at the provincial level (*R*^2^ = 0.977) [22] and hence it provides a timely and inexpensive tool to monitor human settlements, GDP, energy consumption, carbon emissions, and spatial population density [23,24,25]. This encouraged us to produce AHF in China based on the method of Flanner’s study [20]. but replacing population density with nighttime light data.

## 2. Data and Methods

### 2.1. Multi-Temporal Dataset of DMSP-OLS

The DMSP-OLS dataset was acquired from NOAA’s National Geophysical Data Center [26]. The dataset provides cloud-free composites of DMSP-OLS smooth resolution data from 1992 to 2010 [23]. The dataset for stable average nighttime lights are used in this paper, which are given on 30 arc second grids that span −180 to 180 degrees longitude and −65 to 75 degrees latitude. These data (range from 1 to 63) contain the lights from cities, towns, and other sites with persistent lighting, including gas flares. Ephemeral events, such as fires, have been discarded. Areas with zero cloud-free observations were represented by the value 255 [27].

### 2.2. Statistical Data

National primary energy consumption data, including coal, petroleum and natural gas, in China from 1980 to 2012 were provided by the U.S. Energy Information Administration (EIA, available from) [28]. In this paper, we only consider non-renewable energy as this is currently the major resource. Renewable energy makes up only about 5% of energy consumption in China currently, so it is excluded in this study but should be included in future studies as the renewable energy fraction is increasing rapidly. Some primary energy is used to produce secondary energy (for example electricity) in rural areas which is then finally consumed in urban areas. However, it is difficult to calculate the production efficiency and track down the energy transfer. Energy consumption for some specific cities can be downloaded from database of yearly statistics [29]. The urban areas of some cities were provided by the urban statistical yearbook in China [30].

### 2.3. Methods for Calculating AHF

**Figure 1:**
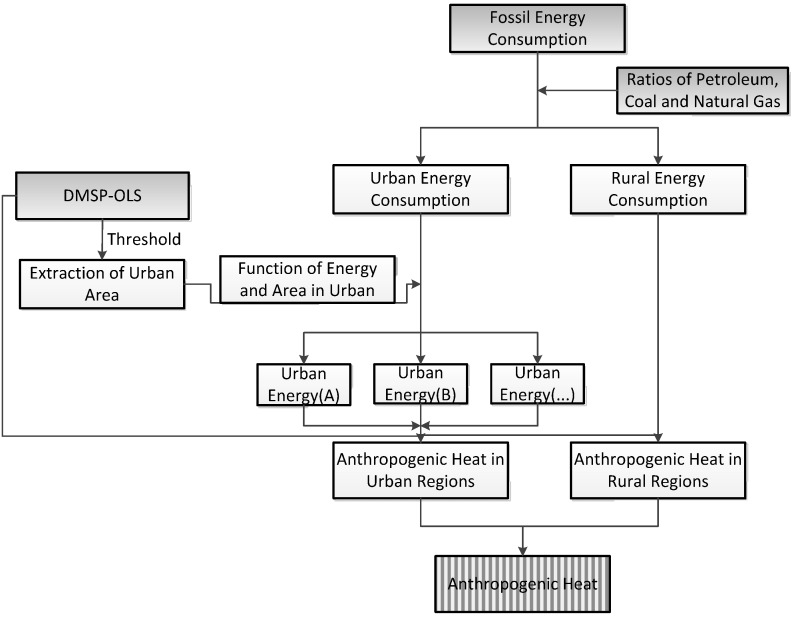
Diagram of estimation for AHF.

**Figure 2:**
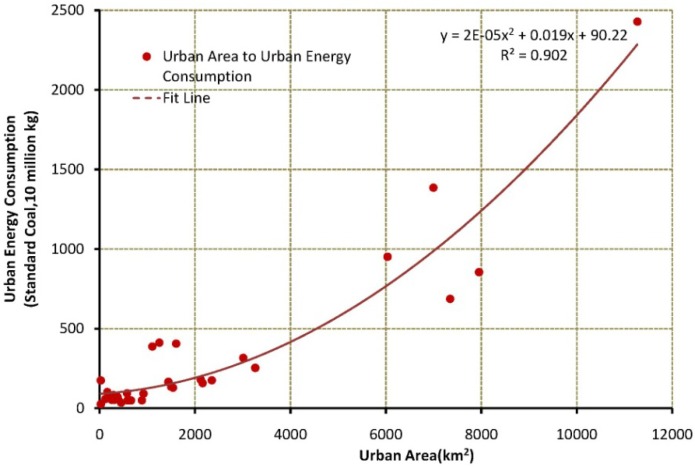
Exponential relationship between urban area and energy consumption by city scale (data of urban areas and energy consumption are provided by NBSC, which is used to fit energy consumption to urban area).

As shown in Figure 1, we used four steps to calculate AHF:

(1) Since larger DMSP-OLS digital number (DN) represents more active economic activities, areas where DN ≥ 12 were classed as urban areas while the remainder are classed as rural regions.
(2) We divide energy consumption (petroleum, coal and natural gas) into two parts: energy used in urban areas and in rural areas. We use the 2009 consumption ratio from IEA (87% coal, 77% oil, and 81% gas for urban energy consumption). This ratio may change annually, but should not change dramatically during the study period.
(3) There exists a strong exponential relationship between urban area and energy consumption (*R*^2^ = 0.9) from the available statistical data (Figure 2). Based on this relationship, energy consumption in the urban area obtained from step (2) is exploited in the urban map obtained in step (1). In China, most large factories or refineries are located in urban areas.Therefore, the energy consumed in urban area not only include the energy consumption for domestic use but also industrial usage. Human metabolism makes up a small share of the total heat released by human activies, and we exclude it here. We note that statistical errors in urban energy consumption are a function of urban area and energy consumption. In order to alleviate their effects, we can allocate total urban energy into patches based on percent-normalized urban energy from Figure 2.
(4) Finally, AHF was calculated using energy data obtained for each urban patch in step (3) according to the percent-normalized DMSP-OLS data in every urban patch and rural region. As data of energy consumption allocated to each grid cell are total energy consumption for corresponding location, the sum consumption is converted to heat flux in each grid (Wm^−2^) by dividing by elapsed time.

## 3. Results

### 3.1. AHF Temporal and Spatial Patterns

**Figure 3:**
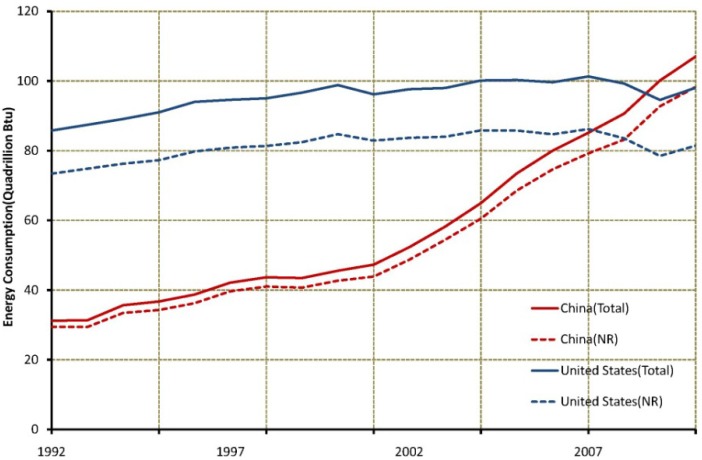
Temporal trends of energy consumption from 1980 to 2010 in China (NR: non-renewable resources, data of energy consumption are provided by EIA. This figure shows temporal trends and differences of energy consumption in America and China from 1992 to 2010).

Energy consumption from 1992 to 2010 increased greatly in China. Total energy consumption in China rose by nearly a factor of 3.42 from 1992 to 2010, and energy consumption in China exceeded the energy consumption in the United States in 2008 (Figure 3). China is projected to contribute the largest share of global energy use in the near future because the energy demand is expected to rise by 60% in 2035 [31]. Non-renewable resources have always contributed the largest share of total energy resources. Compared with clear trends of energy consumption in China during this period, there are no pronounced increasing trends in the United States. Moreover, the difference in average energy consumption between both countries during this period was 47.1 quadrillion Btu.

**Figure 4:**
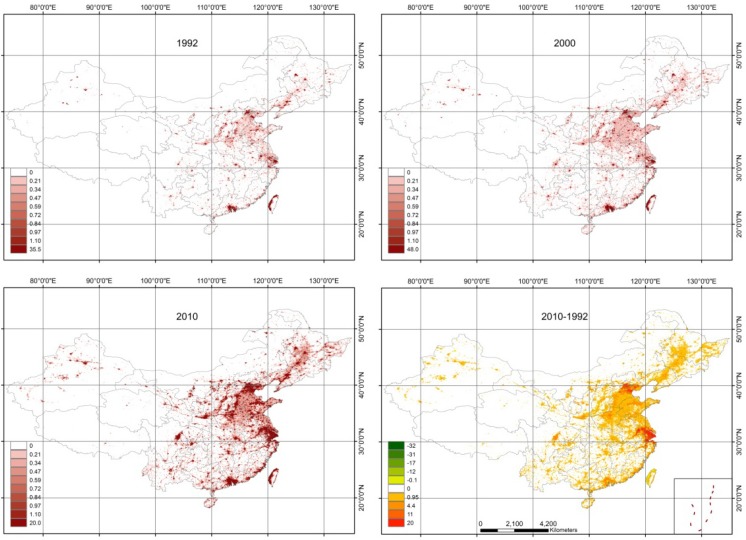
Spatial patterns of AHF and spatial differences of distributions from 1992 to 2010 (units: Wm^−2^).

Figure 4 shows that spatial correlation between AHF distribution and economically developed regions is very close since anthropogenic heat is emitted during the energy consumption process. High AHF is concentrated in megacities, e.g., Beijing, Shanghai and Guangzhou; furthermore, the spatial extent greatly expanded during the studied period. Most of anthropogenic heat concentrated on economical developed regions, while less distributed in developing regions.

AHF in China changed greatly from 1992 to 2010. The increasing trend in anthropogenic heat was focused on three major metropolis: the Beijing-Tianjin region, the Yangzi River delta and the Pearl River delta. The Beijing-Tianjin and the Yangzi River delta had the largest changes during the studied period (Figure 4). The cities of Beijing, Shanghai and Guangzhou had the largest single-city AHF change in the three mega-metropolis. The annual mean AHF in Beijing, Shanghai and Guangzhou were 17, 19 and 7.8 Wm^−2^, respectively, in 2010. However, these values were only 9.0, 7.9 and 6.19 Wm^−2^, respectively, in 1992. The AHF in Shanghai city in 2010 was 2.4 times its size in 1992. On the larger scale, the AHF across China changed obviously between 1992–2000 and 2000–2010; the annual mean AHF for China in 1992, 2000, and 2010 was 0.10, 0.14, and 0.34 Wm^−2^, respectively. Large changes were found during the latter half of the studied period; the AHF difference for China from 2000 to 2010 was 0.2 Wm^−2^. The increase in anthropogenic heat in China was primarily due to acceleration of economic growth.

### 3.2. Validation

It is very difficult to evaluate AHF with *in situ* measurements because identifying whether the sensible or latent heat was emitted from human activity or background conditions is very challenging [32]. Therefore, our data must be validated with other estimated results [33]. This study exploited AHF spatial distributions across China [20], annual average province-level AHF [18,22] and city-scale AHF [9,19] to assess the data.

#### 3.2.1. Comparison of Data for China

**Figure 5:**
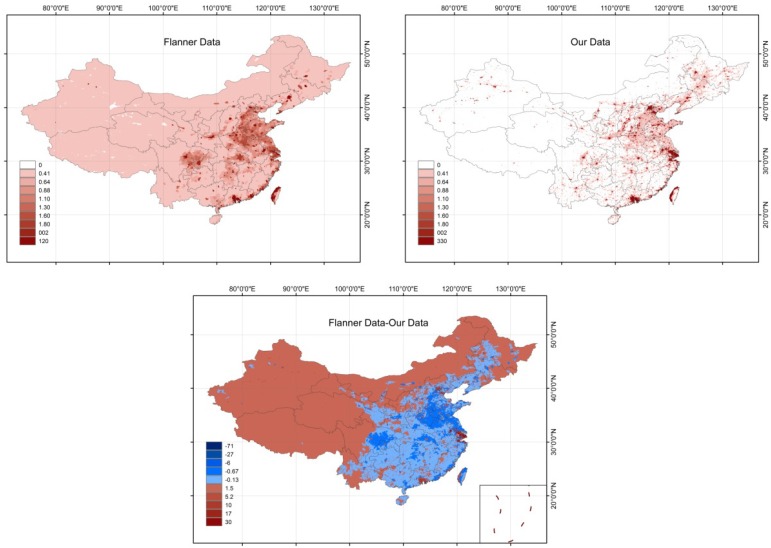
Flanner’s data of AHF, our data and spatial difference of AHF between Flanner and our data in 2005 (2.5 × 2.5 min).

We compare our data with global AHF data [20] (named Flanner’ data), which are estimated by allocating energy consumption by spatial population density. As there are different spatial resolutions for Flanner’s data and our data, we must resample these data to uniform resolution (2.5 × 2.5 min) to compare them. Figure 5 shows that the AHF spatial discrepancies between Flanner’s data and our data are obvious. In urban regions, our data are larger than Flanner’s results, and the largest anthropogenic heat release occurred in mega-cities, such as Shanghai and Beijing. In rural regions, our data are generally small compared with Flanner’s results. In some western and northwest regions in China, the AHF in Flanner’s data are non-zero. However, these regions, such as desert and mountain regions do not consume energy and should have no AHF.

The fact that our data are larger in urban than rural areas is a result of non-renewable energy resources that were allocated to rural regions in Flanner’s analysis and are larger than the actual rural energy consumption. Moreover, accuracy of the spatial population density proxy could result in errors. The annual average AHF estimated by Flanner used yearly energy consumption allocated into spatial population density distributions [20]. AHF data quality depends on the spatial proxy. Spatial population density data were calculated using areas and populations in administration units, resulting in no pronounced statistical spatial change between administration units. The AHF has significant relationships with cities. Moreover, the AHF is inversely proportional to the distances from cities [34]. Therefore, it is unreasonable that there is no change in AHF in certain areas.

#### 3.2.2. Comparison of Province-Level AHF Data

**Figure 6:**
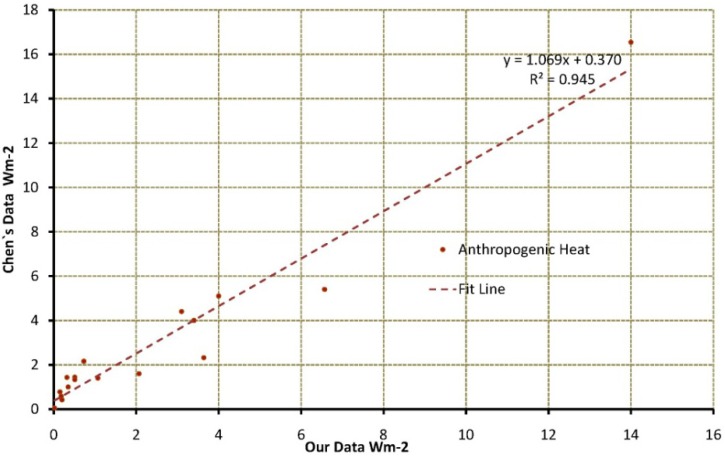
Comparison on AHF between chen’s data and data based on DMSP-OLS.

Annual average province-level AHF from Chen *et al*. were estimated using the energy consumption per province and corresponding areas [18]. Figure 6 shows that there is good consistency between these data; the slope of the curve is 1.6 and correlation coefficient is 0.945. However, there are some deviations, e.g., the maximum AHF difference in Shanxi Province is 0.65 Wm^−2^ and the minimum discrepancy in Xinjiang Province is 0.03 Wm^−2^. Human metabolism was considered in estimating the annual average province-level AHF, which is a relatively small contribution in our data. Although the DMSP-OLS data have a strong linear relationship with energy consumption, the estimated non-renewable resources from a linear model for DMSP-OLS and energy consumption are not equivalent to the actual energy consumptions due to statistical errors, which will produce deviations in some provinces.

#### 3.2.3. Comparison of Single-City AHF Data

The daily AHF for individual cities were acquired to validate our data. Because we used annual average AHF or yearly total energy per pixel, the AHF data needed to be converted to daily AHF using the yearly and daily weight curves of Flanner [17,20]. AHF in Beijing was estimated using the bottom-up method in which vehicles, building density and industry were modeled to estimate AHF. AHF in other cities was estimated using the top-down approach. Table 1 shows that our data have substantial inconsistencies with the AHF for individual cities reported in other studies. The largest difference is 70.23 Wm^−2^ in Beijing; The smallest difference is 25.9 Wm^−2^ in Guangzhou [19]. Comparing Flanner’s, Chen’s and single-city AHF data, our AHF are larger than Flanner’s and Chen’s and smaller than the single-city AHF. Moreover, single-city AHF is more accurate as they consider detailed information for individual cities at smaller scales. Since it is difficult to be access city-level social and economic statistics and the expense in estimating AHF, exploiting bottom-up methods to estimate AHF across China is unrealistic.

**Table 1:** The differences of our data and results of other’s AHF.

Regions	Flanner (Wm^−2^)	Chen (Wm^−2^)	Our Data (Wm^−2^)	AHF of Single City (Wm^−^^2^)	Estimating Methods of Single City
Beijing	-	5.81	24.67	94.9 [19]	Heat Emission Inventory
Guangzhou	7.99	12	15.1	41 [9]	Energy Based
Hangzhou	-	7.84	17.36	50 [35]	Energy Based
Xiaoshan	-	4.62	14.9	40 [35]	Energy Based

The differences between single-city AHF and our data are primarily due to the rules used for allocating total urban energy consumption to individual cities, *i.e.*, the amount of apportioned energy that was consumed in some large cities was smaller than the actual demands. The other reason for this finding is that the daily and yearly weight curves do not depend on climate zones in China. Moreover, inconsistency in the urban areas selected by different authors may result in deviations, especially because too small of an urban area extent leads to high AHF in the corresponding city.

## 4. Discussions

Although anthropogenic heat is only about 0.3% of the total energy transport to the extra-tropics by atmospheric and oceanic circulations, human heat could disrupt the normal atmospheric circulation pattern and bring significant influences on surface temperature at global scales [12]. Therefore, incorporating anthropogenic heat into climate models could improve the performance of simulations of surface climate warming [12,36]. Increased urban heat could possibly reduce concentration of precursor species (e.g., NO_x_ and CO), while increase concentration of surface ozone [36,37], and raise risks of morbidity and mortality [38].

Our analysis shows higher AHF than Flanner’s for urban regions, but smaller than values of studies in specific cities. The applied methodology could be improved in future in several ways. Firstly, our method assumes that all energy is converted into waste heat, which is unreasonable; Secondly, we use only non-renewable energy and the percentage of renewable energy is increasing; Thirdly, we use primary energy statistics not the final energy consumption in each location, which will exclude the waste heat released in the production process; Lastly, threshold and saturation in the DMSP-OLS data may make it difficult to monitor anthropogenic activity in mega-cities [39].

## 5. Conclusions

We allocated annual fossil energy consumption to DMSP-OLS data from 1992 to 2010 in China using the following procedure: (1) divide total energy consumption into urban and rural components; (2) apply threshold (digital number ≥ 12) to distinguish urban and rural regions based on the DMSP-OLS data; (3) allocate energy consumption of urban area into each city based on the relationship between the urban area and energy consumption records from several specific Chinese cities; and (4) calculate AHF based on DMSP-OLS data for urban and rural areas separately.

Our results show that AHF increased greatly in China from 1992 to 2010. The biggest changes occurred in the Beijing-Tianjin region, the Yangzi River delta and the Pearl River delta. During the latter half of the studied period, AHF across China increased due to booming economic development. Compared with other data, our results show reasonable performance with better spatial distribution and larger magnitude for large cities than that in Flanner data. This illustrates that the proxy of DMSP-OLS is better than population density for AHF studies as it represents economic activity.
